# Previously Incarcerated Veteran: A Case of Drug-Induced Parkinsonism

**DOI:** 10.7759/cureus.98525

**Published:** 2025-12-05

**Authors:** Brian R Bailey, Ashley Mason, Vien Dinh

**Affiliations:** 1 Emergency Medicine, Nova Southeastern University Dr. Kiran C. Patel College of Osteopathic Medicine, Clearwater, USA; 2 Neurology, University of South Florida Morsani College of Medicine, Tampa, USA; 3 Psychiatry, Veterans Affairs Medical Center, St. Petersburg, USA

**Keywords:** aripiprazole, benztropine, drug-induced parkinsonism, extrapyramidal symptoms, incarceration, risperidone, schizophrenia, second-generation antipsychotics, veterans

## Abstract

Drug-induced Parkinsonism (DIP) is a recognized extrapyramidal side effect (EPS) associated with prolonged antipsychotic therapy, and typically occurs early in antipsychotic initiation. We present a rare case of late-onset DIP in a 64-year-old previously incarcerated male veteran with schizophrenia, who had been maintained on risperidone for nearly 40 years without regular psychiatric follow-up. The patient presented with a one-month progression of rigidity, dysarthria, and dysphagia. Upon admission, neurological evaluation revealed significant cogwheel rigidity, slow speech, flat affect, and dysdiadochokinesia consistent with DIP. Treatment involved gradual tapering of risperidone, introduction of benztropine for symptomatic relief, and initiation of aripiprazole to maintain psychiatric stability. Symptoms markedly improved, reaffirming the diagnosis of DIP over idiopathic Parkinson's disease. This case emphasizes the importance of regular medication review and follow-up in managing antipsychotic therapy to prevent severe EPS complications, particularly in vulnerable populations such as incarcerated individuals and military veterans.

## Introduction

The use of antipsychotic medications for the treatment of schizophrenia is well-established; second-generation antipsychotics (SGAs) are preferred as they improve both positive and negative symptoms with a reduced side-effect profile compared with first-generation antipsychotics [1,2]. Antipsychotics exert their effects through dopamine D2 receptor blockade in the brain's nigrostriatal pathway, which regulates motor control; second-generation antipsychotics (SGAs) bind these receptors with lower affinity than first-generation agents, thereby reducing but not eliminating the risk of D2 receptor-related side effects [1-5]. This risk of movement-associated side effects, called extrapyramidal side effects (EPS), also rises in patients taking multiple antipsychotics simultaneously [2,6]. EPS includes dystonias, tardive dyskinesia, and, importantly, Parkinsonism [4,7,8]. Parkinson's disease (PD) itself is a progressive neurodegenerative disorder characterized by the loss of dopaminergic neurons in the substantia nigra pars compacta and the accumulation of misfolded α-synuclein aggregates (Lewy bodies and neurites) that disrupt neuronal and synaptic function [9,10]. The pathogenesis involves mitochondrial dysfunction, lysosomal impairment, neuroinflammation, and aberrant protein aggregation, with genetic mutations in SNCA, LRRK2, PINK1, and Parkin providing mechanistic insight into both familial and sporadic disease [10]. Experimental models have been critical to discovering these mechanisms: neurotoxin-based animal models (e.g., MPTP, 6-OHDA, rotenone) and pharmacologic models (e.g., haloperidol-induced catalepsy) reproduce core motor and pathological features of PD and drug-induced Parkinsonism [11-13]. Acute dopamine receptor blockade in the substantia nigra pars reticulata produces neuronal firing patterns and motor deficits analogous to those seen in dopamine-depleted states [12,14]. These findings establish biological plausibility for drug-induced Parkinsonism (DIP) and demonstrate how various inducing agents, including antipsychotics, calcium-channel antagonists, and environmental toxins, affect dopaminergic signaling and motor function [11,13].

DIP is characterized by classical Parkinson's features-rigidity, dysarthria, tremor, and balance difficulties- and can be mistaken for idiopathic Parkinson's disease due to its similarity and insidious onset [15]. Patients receiving long-term antipsychotic therapy are at increased risk for its development [4,15]. In clinical studies, EPS onset has been shown to peak at three months and then again at 12 months post-antipsychotic initiation, for some patients showing up in days to weeks [5]. One case describes DIP occurring after 10 years of risperidone, although this is uncommon [7]. 

In addition to EPS, second-generation antipsychotics carry a black-box warning for increased mortality in patients with dementia-related psychosis [8]. Because of these adverse events, current American Psychiatric Association (APA) guidelines recommend that patients be monitored regularly [16]. Consistent monitoring is challenging in populations frequently lost to follow-up (LTFU). Incarcerated patients are one such group: one study found that two-thirds of inmates are LTFU immediately after release and, of those who do follow up, 39% are seen only once [17]. Veterans are also often LTFU and may miss essential medications [18]. Consequently, unmanaged antipsychotic therapy poses a substantial risk for the development of EPS in these populations [1,4,16].

This case describes a patient treated with risperidone who presented with Parkinsonian symptoms after being LTFU for nearly 40 years, and whose symptoms started decades after SGA initiation.

## Case presentation

The patient is a 64-year-old male military veteran with a medical history of hypertension, hyperlipidemia, type 2 diabetes mellitus, and schizophrenia, the latter diagnosed during incarceration approximately 40 years ago. He presented for evaluation and management of a one-month history of left upper extremity stiffness, dysarthria, and dysphagia. Following admission, he was evaluated by the inpatient psychiatry consultation team for these symptoms.

Past psychiatric history

The patient had a roughly 40-year history of schizophrenia managed with risperidone 6 mg daily (2 mg in the morning, 4 mg at night), Depakote (500 mg each morning, 750 mg each night), and hydroxyzine 50 mg daily. Prior to admission, he had been taking these medications consistently without psychiatric outpatient follow-up. Three to four months prior to admission, his primary care physician started him on mirtazapine 45 mg nightly.

He was initially diagnosed with schizophrenia in 1989, shortly after military discharge and upon incarceration. Prior to diagnosis, he reported auditory hallucinations commanding him to harm himself and others, which ultimately led to the crime resulting in his incarceration. He also reported significant paranoia and sleep disturbances at that time.

Social history

The patient stated his schizophrenia had been well-controlled over the past 40 years and reported living independently at home until the recent onset of physical symptoms. He described his sleep as well-managed on his psychiatric medication regimen.

Timeline of present illness

The patient described a one-month progressive difficulty lifting objects, such as a remote control, with his left hand. One week prior to admission, he noticed increasing difficulty walking and generalized slowing of movements. Four days prior to admission, he developed difficulties with speech and swallowing, affecting both solids and liquids. He denied experiencing anosmia, constipation, or symptoms suggestive of orthostatic hypotension.

Initial physical examination on admission

The patient is an elderly African American male, appearing consistent with his stated age. He was pleasant and cooperative but made poor eye contact. His speech was slow, slurred, and of decreased volume. His mood was neutral and affect flat. Thought processes were periodically tangential but redirectable. He denied auditory or visual hallucinations. He was alert and oriented to person, place, time, and situation, with adequate attention. Both short-term and long-term memory appeared grossly intact.

Cranial nerves II-XII were equal and intact bilaterally. No facial drooping was observed. Motor strength was grossly intact bilaterally in the upper and lower extremities. The patient demonstrated significant rigidity bilaterally in the upper extremities and had difficulty performing rapid alternating movements such as toe and finger tapping. Mild intention tremor was noted bilaterally during the finger-to-nose test. His gait was broad-based and shuffling, with difficulty turning.

Differential diagnoses and management

The patient's clinical presentation raised significant concern for drug-induced Parkinsonism versus idiopathic Parkinson's disease. His history of subacute-onset rigidity in the context of prolonged antipsychotic use strongly suggested drug-induced Parkinsonism. Consequently, the patient's antipsychotics were gradually tapered, and benztropine was introduced for symptomatic management. The patient demonstrated significant improvement following these medication adjustments (see Table 1 for medication changes), with complete resolution of hypomimetic speech and dysphagia and notable improvement in cogwheel rigidity by the time of discharge. Due to this clinical improvement following changes in his antipsychotic regimen, idiopathic Parkinson's disease was considered significantly less likely, and further diagnostic testing, such as dopamine transporter (DAT) scanning, was not pursued.

**Table 1:** Changes in medication regimen and subsequent changes in examination over hospitalization At the start of hospitalization, we discontinued the patient's home Divalproex sodium. Valproate levels were 59.0 on admission. Patient was continued on mirtazapine 45 mg PO QHS and hydroxyzine 50 mg PO daily throughout admission. QHS - nightly; QAM - every morning; BID - twice daily; PO - oral pills/ by mouth; DIP - drug-induced Parkinsonism

Day	Current Medications	Intervention	Reason for Intervention	Clinical Response
1	Risperidone 2 mg in morning, 4 mg at night.	Added Benztropine 0.5 mg daily. Downtitrated risperidone to 4 mg daily (2 mg BID). Continued home mirtazapine and hydroxyzine.	Benztropine assists with drug-induced Parkinsonism. Downtitrating antipsychotics is first-line for DIP.	Patient reported mild improvement in speech overnight; flat affect still present.
2	Benztropine 0.5 mg daily. Risperidone 2 mg BID.	Decreased risperidone to 1 mg in the morning, 2 mg nightly.	Patient continuing to experience DIP symptoms.	Patient with improvement in flat affect and cognition.
3	Benztropine 0.5 mg daily. Risperidone 1 mg in the morning, 2 mg at night.	Increased benztropine to 1 mg daily. Provided lorazepam 0.5 mg.	Benztropine can be uptitrated when still with severe motor abnormalities such as rigidity. Lorazepam is second line.	Patient with mild improvement in dysphagia and left arm weakness.
4	Benztropine 1.0 mg daily. Lorazepam 0.5 mg once. Risperidone 1 mg in the morning, 2 mg at night. Depakote 500 mg in morning, 750 mg at night.	Provided lorazepam 0.5 mg twice. Patient missed two doses of risperidone overnight due to declining medication.		Same as the prior day.
5	Benztropine 1.0 mg daily. Lorazepam 0.5 mg twice. Risperidone 1 mg in the morning, 2 mg at night (however patient with two missed doses).	Decreased risperidone to 0.5 mg every morning and 2 mg nightly. Provided lorazepam 0.5 mg. Added aripiprazole 5 mg every morning.	Patient improvement with risperidone changes thus far.	Overnight, the patient reported mild auditory hallucinations before receiving the evening dose of risperidone.
8	Benztropine 1.0 mg daily. Lorazepam 0.5 mg once. Risperidone 0.5 mg in the morning, 2 mg at night. Aripiprazole 5 mg every morning.	Decreased risperidone to 2 mg QHS.		Patient remained without any hallucinations and reported significant improvement in speech and swallowing.
9	Benztropine 1.0 mg daily. Risperidone 2 mg at night. Aripiprazole 5 mg every morning.	Discharged home on: Risperidone 2 mg QHS, Aripiprazole 5 mg QAM, Mirtazapine 45 mg PO QHS, Benztropine 1 mg PO Daily, Hydroxyzine 50 mg PO Daily.		Patient states he only has mild rigidity in arms left arm, has had good sleep, and improvement in his overall cognition.

## Discussion

This case demonstrates effective treatment for DIP and emphasizes the importance of regular follow-up in patients receiving long-term antipsychotic therapy. After approximately 40 years on the maximum dose of risperidone, this patient developed cogwheel rigidity, speech alterations, and dysdiadochokinesia.

Determination of diagnosis

The initial step in treatment involved gradual discontinuation of the offending agent, risperidone, consistent with APA guidelines that recommend withdrawal of antipsychotics upon the development of extrapyramidal symptoms (EPS) [16]. Gradual tapering was chosen to prevent exacerbation of the underlying schizophrenia and resulted in partial symptomatic improvement. Removing the causative agent is a common method used to distinguish drug-induced Parkinsonism from idiopathic Parkinson's disease, as is the absence of anosmia [1,19]. However, in some cases, symptoms do not improve after drug withdrawal, necessitating further diagnostic imaging to assess for underlying Parkinsonism unrelated to antipsychotic use [19].

Additionally, neurological consultation is essential to rule out Parkinson's disease and other neurological etiologies. This multidisciplinary approach ensures a comprehensive assessment. In this case, neurology concluded that the patient's symptoms were drug-induced, recommending continued psychiatric management.

Treatment of Parkinsonian symptoms

Benztropine was initiated to manage the EPS while risperidone was gradually tapered. Benztropine, an antimuscarinic medication with anticholinergic properties, is commonly employed to alleviate rigidity in drug-induced Parkinsonism [7,16].

Aripiprazole was introduced to replace risperidone for schizophrenia management due to its lower risk of inducing Parkinsonism [3,20] and its efficacy against positive schizophrenic symptoms [20]. This is theoretically attributed to aripiprazole's unique pharmacological profile, combining dopamine D2 receptor agonism and antagonism, stabilizing dopamine signaling by preventing both excessive blockade and excessive stimulation [3,20].

Cross-titration of risperidone and aripiprazole successfully controlled schizophrenic symptoms while improving the patient's Parkinsonian symptoms. Cross-titration helps prevent lapses in symptom control and minimizes prolonged hospitalization [21,22]. Throughout hospitalization, the patient experienced only one episode of visual hallucinations after missing a risperidone dose, shortly before initiating aripiprazole on day four. Medication adjustments paused on day seven as the patient's symptoms had largely resolved, and preparations for discharge began. Completion of cross-titration was planned on an outpatient basis, as transition to monotherapy is generally well-tolerated, reducing mortality and morbidity among schizophrenia patients [6], particularly when transitioning to aripiprazole [21]. Outpatient completion may not suit every patient; however, this patient had robust family support, good insight, and planned psychiatric follow-up.

These medication adjustments offer clinicians a potential strategy to reduce polypharmacy in schizophrenia management. Aripiprazole specifically has demonstrated effectiveness in symptom control, including hallucinations, as illustrated by this patient [20,21]. Clozapine represents another alternative with minimal EPS risk, comparable to placebo, that clinicians might consider as a substitute for risperidone [23].

Bridging antipsychotics, as demonstrated in this case, can prevent psychotic relapse in previously stable patients [21,24], especially with the use of aripiprazole [21].

Consequences of patients lost to follow-up

Although DIP is a known complication of antipsychotic therapy [4,19], its occurrence in a patient stable on medication for nearly 40 years is notable, as numerous studies show it occurring within the first year to decade of medication initiation [5,7]. This underscores the critical need for consistent patient follow-up, particularly within vulnerable populations such as formerly incarcerated individuals and military veterans [17,18].

In this case, the patient continued receiving antipsychotics initiated during incarceration without psychiatric oversight, allowing Parkinsonian symptoms to progress to the point of dysphagia and impaired daily functioning.

Importance of regular assessment in schizophrenia

This patient's symptoms emerged after decades of treatment, highlighting the necessity of routine monitoring and drug safety assessment even in long-term stable patients. Despite being considered low-risk for EPS, risperidone-associated EPS occurs in 70.2% of patients taking it, similar to how this patient developed it [25]. While DIP typically emerges earlier in treatment, this case demonstrates that even patients who appear stable for decades remain vulnerable. This case exemplifies the APA recommendations advocating ongoing follow-up and consistent screening in schizophrenia management [7,16].

## Conclusions

Drug-induced Parkinsonism is a common complication associated with antipsychotic therapy. Patients with histories of incarceration or military service represent vulnerable populations who may remain untreated or inadequately managed for prolonged periods. Clinicians can mitigate these risks by regularly reassessing medication regimens and safely transitioning patients to newer antipsychotics with a lower risk of drug-induced Parkinsonism, even when psychotic symptoms have been stable on long-standing treatments.
